# A Case Report of Premalignant Plasma Cell Dyscrasia-Induced Renal Failure in a 31-Year-Old Female

**DOI:** 10.1155/2022/2497380

**Published:** 2022-06-07

**Authors:** Ayrton Bangolo, Mahabuba Akhter, Amer Jarri, Manpreet Kaur, Ali Atoot, Parul Jandir, Mahmood Ibrahim, Lochana Manandhar, Adam Atoot

**Affiliations:** ^1^Hackensack, Meridian Health Palisades Medical Center, Department of Internal Medicine, North Bergen, NJ, USA; ^2^Hackensack University Medical Center, Department of Anesthesia, Hackensack, NJ, USA; ^3^Hackensack Meridian Health Palisades Medical Center, Department of Nephrology, North Bergen, NJ, USA

## Abstract

Monoclonal gammopathy of renal significance (MGRS) is a rare disorder in which monoclonal immunoglobulin secreted by nonmalignant B cell or plasma cell clone causes kidney damage. Although MGRS is a premalignant condition, it can cause severe kidney disease and end-stage renal disease (ESRD) at any age. Herein, we present a 31-year-old female with past medical history of lupus nephritis who presented with signs of volume overload and worsening renal function despite adequate immunosuppressive therapy. Renal biopsy revealed heavy and light chain deposition consistent with MGRS. This case report demonstrates the importance of including MGRS in the differential diagnosis of worsening renal function despite adequate treatment, raising awareness of this premalignant yet morbid condition.

## 1. Introduction

Monoclonal gammopathy of undetermined significance (MGUS) consists of a premalignant clonal plasma cell or lymphoplasmacytic proliferative disorder. MGUS is characterized by the absence of end organ damage [1]. However, some patients, meeting diagnosis criteria of MGUS can develop significant kidney injury, and the condition is then termed monoclonal gammopathy of renal significance (MGRS) [2]. MGRS is associated with high morbidity due to the severity of renal lesions. Suppression of monoclonal immunoglobulins (MIg) often improves outcome [3]. Here, we report the case of a 31-year-old female with stage 4 lupus nephritis and worsening renal function despite adequate treatment, found to have MIg on renal biopsy. With this case report we aim to encourage physicians to include MGRS in the differential diagnosis of rapidly progressing renal failure, as early detection and intervention will improve the outcome.

## 2. Case Presentation

This is a 31-year-old female with past medical history significant for stage IV lupus nephritis who presented for evaluation of a 2-day history of worsening face and extremities swelling. The patient denied any recent insect bite, joint pain, hematuria, or recent upper respiratory tract infection. Of note, the patient was evaluated a year ago for the same symptoms and was found to have severe renal dysfunction. A renal biopsy at that time revealed stage IV lupus nephritis. The patient was discharged on prednisone and mycophenolate. However, she reports noncompliance to the regimen.

Physical exam was notable for facial swelling, bilateral jugular venous distention, and 3+ bilateral lower extremity edema. Laboratory results revealed a creatinine of 7.58 (0.6–1.2) milligram per deciliter (mg/dL), Creatinine a year ago upon discharge was 1.21 mg/dL. She was also found to be hyponatremic, with low serum protein, elevated serum free kappa and lambda light chains, and normal kappa/lambda ratio as seen in Table 1.

The patient was started back on prednisone and mycophenolate which she had self-discontinued; nephrology was consulted as well. The patient's creatinine further deteriorated despite being on the regimen. A serum electrophoresis revealed a visible faint band with polyclonal pattern in the gamma region, a developing plasma cell disorder could not be excluded as seen in Table 2. A subsequent serum immunofixation revealed a faint band in immunoglobulin G (IgG) and lambda chain against a dense polyclonal background.

**Figure 1:**
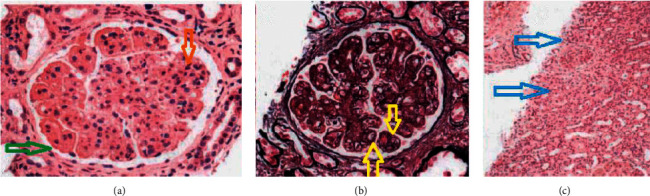
Image (a) showing diffuse proliferative glomerulonephritis, reduced bowman space (green arow) and increased cellularity in glomeruli (orange arow). Image (b) showing global glomerular basement membrane duplication (yellow arrow). Image (c) showing cortical scarring (blue arow).

**Figure 2:**
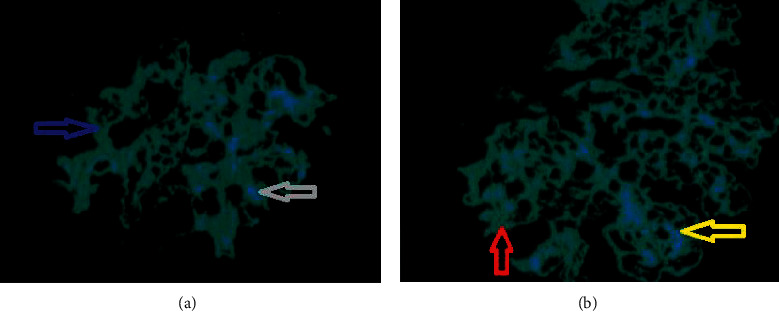
Image (a) showing immunofluorescence (IF) staining for immunoglobulin G 3 (IG3), green representing the signal for IG3 (blue arow), with the nuclei in blue pointed with the gray arow. Image (b) showing IF staining for lambda, green representing the signal (red arow), with the nuclei in blue pointed with the yellow arow.

The patient was continued on prednisone and mycophenolate, and furosemide was added to the regimen with some improvement of the volume overload. She underwent a renal biopsy which revealed diffuse proliferative glomerulonephritis with prominent membranoproliferative features as seen on Figure 1. Immunofluorescence revealed granular, global mesangial, and capillary wall deposits each staining 3 + for IgG-3, complement 3, complement 1, and lambda as seen on Figure 2. Findings were consistent with proliferative glomerulonephritis with monoclonal IgG deposits, and a diagnosis of monoclonal gammopathy of renal significance was then made. The patient was started on bortezomib, cyclophosphamide, and prednisone was switched to dexamethasone, and mycophenolate was discontinued.

## 3. Discussion

Monoclonal gammopathy of renal significance (MGRS) encompasses patients who would otherwise meet the criteria for monoclonal gammopathy of undetermined significance (MGUS) but demonstrate kidney injury attributable to the underlying monoclonal protein [2]. Monoclonal gammopathy is more frequent in systemic lupus erythematosus (SLE) patients than in the general population [4]. SLE may present with nephritis as the sole disease manifestation [5]. In one study by Giavinti et al. in which nephritis was the initial manifestation of SLE, antinuclear (ANA) and anti-double-stranded DNA antibodies were positive up to 10 years after the original presentation [6]. Our patient was diagnosed, a year ago, with lupus nephritis. However, she did not have any clinical manifestations of SLE, and the ANA and anti-double-stranded DNA antibodies were negative. Given her history of lupus nephritis, the patient was at higher risk of developing MGRS.

In monoclonal gammopathy of renal significance (MGRS), the kidney lesions are primarily caused by the abnormal deposition or activity of monoclonal proteins in the kidney [7]. Monoclonal proteins can also act as autoantibodies directed against complement components, leading to uncontrolled activation of the alternative complement pathway, and causing complement 3 (C3) glomerulopathy [8]. Cases of monoclonal anti-glomerular basement membrane (GBM) disease due to circulating monoclonal antibodies have been reported [9, 10]. Monoclonal deposits must be restricted to a single class of light and/or heavy chain based on immunofluorescence to make the diagnosis of MGRS [11]. For unclear reasons, most patients with proliferative glomerulonephritis with monoclonal immunoglobulin deposits (PGNMID) do not have detectable circulating monoclonal gammopathy by serum and urine monoclonal protein testing [12]. Our patient's renal biopsy revealed monoclonal deposits of a single class of light chain (lambda) and a single class of heavy chain (immunoglobulin G) consistent with MGRS. The biopsy also revealed C3 deposit, consistent with C3 glomerulopathy, and a duplication of GBM was also reported. The patient's biopsy was consistent with proliferative glomerulonephritis with prominent membranoproliferative features and the serum electrophoresis did not reveal a monoclonal spike, and the immunofixation only revealed a faint immunoglobulin G and lambda. These findings are adequate with the literature.

Patients with monoclonal gammopathy-associated proliferative glomerulonephritis (PGNMID or C3 glomerulopathy with monoclonal gammopathy) are at risk for progressive kidney disease and should be treated to prevent further kidney injury and deterioration of kidney function. The treatment focuses upon eradication of the pathologic clone [13]. In patients with a detectable plasma cell clone, a treatment regimen similar to that used to treat multiple myeloma (MM) is recommended [13]. Treatment should be continued for up to six months if there is evidence of a hematologic response and no toxicity [13]. Our patient's biopsy expressed plasma cell monoclonal protein, with a heavy chain (immunoglobulin G) complexed with a light chain (lambda). She qualified for a treatment regimen similar to that used in MM. The patient will be reevaluated for response to treatment at regular intervals.

## 4. Conclusion

Monoclonal gammopathy of renal significance (MGRS) is a premalignant clonal plasma cell or lymphoplasmacytic proliferative disorder. Although MGRS is a premalignant condition, it is associated with high morbidity due the rapidly progressive nature of kidney disease. Early detection is essential, as suppression of immunoglobulin improves the outcome. With this case report, we hope to encourage clinicians to include MGRS in the differential diagnosis of unexplained worsening kidney function and raise awareness of this premalignant yet morbid condition.

## Figures and Tables

**Table 1:** Laboratory values on admission and a year ago.

	Laboratory values
Creatinine upon admission	7.58 (0.6–1.2) milligram per deciliter (mg/dL)
Creatinine a year ago upon discharge	1.21 mg/dL
Blood urea nitrogen	38 (7–25) mg/dL
Total serum protein	4.5 (6.4–8.9) gram per deciliter (g/dL)
Serum albumin	2.2 (3.5–5.7) g/dL
Sodium	129 (136–145) millimole per liter (mmol/L)
Calcium	6.8 (8.6–10.3) mg/dL
24-hours total urine protein	6456 mg/24 hours (<150 mg/24 hours)
Serum complement 3	56 (83–193) mg/dL
Serum complement 4	24 (15–57) mg/dL
Free kappa light chain in the serum	44.9 (3.3–19.4) milligram per liter (mg/L)
Free lambda light chain in the serum	47.1 (5.7–26.3) mg/L
Kappa/lambda light chain free ratio	0.95 (0.26–1.65)
Antinuclear antibody	Negative
Double-stranded DNA antibody	Negative

**Table 2:** Serum protein electrophoresis with immunofixation.

	Laboratory values
Total protein	3.9 (6.1–8.1) gram per deciliter (g/dL)
Albumin	2 (3.8–4.8)
Alpha 1 globulin	0.3 (0.2–0.3)
Alpha 2 globulin	0.8 (0.5–0.9)
Beta 1 globulin	0.3 (0.4–0.6)
Beta 2 globulin	0.2 (0.2–0.5)
Gamma globulin	0.4 (0.8–1.7)
Abnormal protein band	Faint band visible with overall polyclonal pattern in the gamma region, a developing plasma cell disorder cannot be excluded
Serum immunofixation	A faint band in immunoglobulin *G* and lambda against a dense polyclonal background. a developing plasma cell disorder cannot be excluded

## Data Availability

All the data generated or analyzed during this study are available from the corresponding author upon request.

## References

[1] Rajkumar S. V., Dimopoulos M. A., Palumbo A. (2014). International Myeloma working group updated criteria for the diagnosis of multiple myeloma. *The Lancet Oncology*.

[2] Leung N., Bridoux F., Hutchison C. A. (2012). Monoclonal gammopathy of renal significance: when MGUS is no longer undetermined or insignificant. *Blood*.

[3] Bridoux F., Leung N., Leung N. (2015). Diagnosis of monoclonal gammopathy of renal significance. *Kidney International*.

[4] Ali Y. M., Urowitz M. B., Ibanez D., Gladman D. D. (2007). Monoclonal gammopathy in systemic lupus erythematosus. *Lupus*.

[5] Adu D., Williams D. G., Taube D. (1983). Late onset systemic lupus erythematosus and lupus-like disease in patients with apparent idiopathic glomerulonephritis. *Quarterly Journal of Medicine*.

[6] Gianviti A., Barsotti P., Barbera V., Faraggiana T., Rizzoni G. (1999). Delayed onset of systemic lupus erythematosus in patients with “full-house” nephropathy. *Pediatric Nephrology*.

[7] Sayed R. H., Wechalekar A. D., Gilbertson J. A. (2015). Natural history and outcome of light chain deposition disease. *Blood*.

[8] Meri S., Koistinen V., Miettinen A., Törnroth T., Seppälä I. J. (1992). Activation of the alternative pathway of complement by monoclonal lambda light chains in membranoproliferative glomerulonephritis. *Journal of Experimental Medicine*.

[9] Turner M., Crawford A., Winterbottom C. (2020). Heavy chain deposition disease presenting with raised anti-GBM antibody levels; a case report. *BMC Nephrology*.

[10] Coley S. M., Shirazian S., Radhakrishnan J., D’Agati V. D. (2015). Monoclonal IgG1*κ* anti-glomerular basement membrane disease: a case report. *American Journal of Kidney Diseases*.

[11] Klomjit N., Leung N., Fervenza F., Sethi S., Zand L. (2020). Rate and predictors of finding monoclonal gammopathy of renal significance (MGRS) lesions on kidney biopsy in patients with monoclonal gammopathy. *Journal of the American Society of Nephrology*.

[12] Fish R., Pinney J., Jain P. (2010). The incidence of major hemorrhagic complications after renal biopsies in patients with monoclonal gammopathies. *Clinical Journal of the American Society of Nephrology*.

[13] Sethi S., Rajkumar S. V. (2013). Monoclonal gammopathy-associated proliferative glomerulonephritis. *Mayo Clinic Proceedings*.

